# Iatrogenic Facial Nerve Injury in Head and Neck Surgery in the Presence of Intraoperative Facial Nerve Monitoring With Electromyography: A Systematic Review

**DOI:** 10.7759/cureus.48367

**Published:** 2023-11-06

**Authors:** Jaswanthi Dogiparthi, Smaran S Teru, Christine M Lomiguen, Justin Chin

**Affiliations:** 1 Medical Education, Lake Erie College of Osteopathic Medicine, Erie, USA; 2 Family Medicine, Lake Erie College of Osteopathic Medicine Health Millcreek Community Hospital, Erie, USA; 3 Pathology, Lake Erie College of Osteopathic Medicine, New York, USA; 4 Family Medicine, LifeLong Medical Care, Richmond, USA

**Keywords:** facial nerve injury, iatrogenic facial nerve injury, ifnm, cochlear implant, vestibular schwannoma, electromyography, nerve monitoring, parotid surgery, facial nerve paralysis, facial nerve

## Abstract

The facial nerve is the seventh of 12 cranial nerves found in the head and neck region that facilitates several nerve fibers and pathways to perform various functions. Iatrogenic facial nerve injury during surgeries of the head and neck is common, ranging from 4-6%, particularly in procedures that involve mobilization or resection of associated anatomical structures. Any injury to the facial nerve or its branches impacts the quality of life and patient satisfaction as the degree of iatrogenic injury may result in partial or complete facial nerve paralysis. Of the various implementable techniques available to avoid injury, electromyography (EMG) has recently been widely used to monitor facial nerve function intraoperatively to determine the degree of injury and predict postoperative weakness. The purpose of this study was to analyze and review existing scientific literature in determining the role of intraoperative facial nerve monitoring (IFNM) with EMG in decreasing the incidence and degree of intraoperative facial nerve injury among commonly performed surgeries involving the facial nerve.

A systematic review was conducted from articles published between September 2006 and December 2022. Suitable articles were identified from the MEDLINE/PubMed databases using relevant terms to meet the inclusion criteria. Articles were subsequently coded based on the inclusion/exclusion criteria as well as the type of surgery performed with concurrent use of EMG and the results from intraoperative monitoring. A total of 47 articles were found in relation to the use of IFNM, including studies to reduce the incidence and determine preventative measures to decrease nerve injury. Eleven articles were used to evaluate the use of EMG during various head and neck surgeries in decreasing the incidence of intraoperative facial nerve injury.

Sources found were primarily divided based on the type of surgery performed when determining the use of EMG. Four sources tested the efficacy of EMG during parotidectomy, four sources during vestibular schwannoma resection, two sources during cochlear implant surgeries, and one during a lymphatic malformation surgery. IFNM also decreased the duration of surgery, the severity of facial nerve palsy, and the average time of facial nerve paralysis recovery. IFNM was found to not significantly predict facial nerve injury in the setting of intraoperative nerve injury but tended to preserve potential facial nerve function in vestibular schwannoma cases.

The surgical setting determined the efficacy and use of IFNM in decreasing the incidence of facial nerve weakness and paralysis. IFNM had the best preventative and prognostic value when used in vestibular schwannoma resection, and the least in cochlear implants, with mixed evidence seen in the setting of parotidectomy. Overall, IFNM using EMG as an adjunct during surgery may reduce the risk of iatrogenic injury; however, additional studies must be performed to determine the degree of long-term patient satisfaction and quality of life achieved in the setting of IFNM.

## Introduction and background

The facial nerve provides innervation to the facial muscles within the head and neck region. As the seventh cranial nerve, it carries motor, sensory, and parasympathetic nerve fibers with general somatic efferent, general visceral efferent, special visceral afferent, and general somatic afferent functions [1,2]. The motor pathway is responsible for innervating muscles of facial expression, volume modulation, and contributions to other accessory neck movements [3,4]. The parasympathetic pathway is controlled by the greater petrosal and chorda tympani nerves, which cause secretion at the lacrimal and submandibular/sublingual glands, respectively [5,6]. The special sensory pathway conveys information about taste to the anterior two-thirds of the tongue by the chorda tympani branch of the facial nerve [7]. Given its complexity, the branches of the facial nerve require surgical finesse with dissection and surgical manipulation.

**Figure 1:**
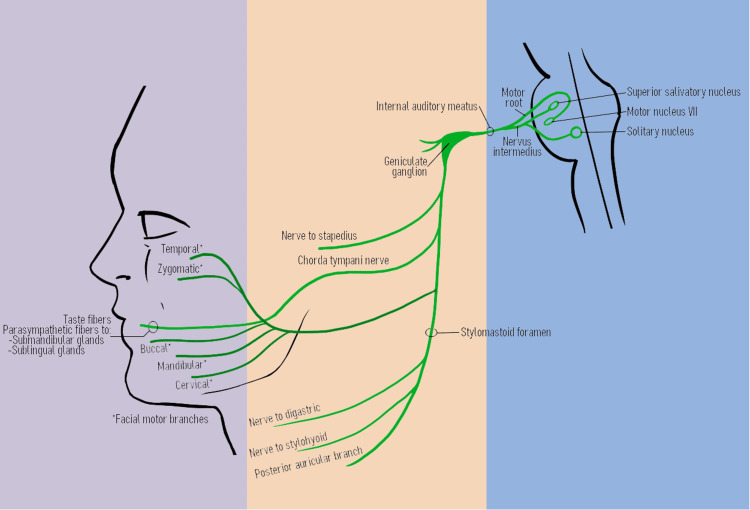
Diagram of the facial nerve with a delineation between intracranial (blue), intratemporal (orange), and extratemporal (purple) regions. Medical illustration by Katherine Pan.

Apart from its function, the facial nerve can further be divided into intracranial, intratemporal, and extratemporal parts (Figure 1). The intracranial portion of the facial nerve courses through the internal auditory meatus in the temporal bone to go through the facial canal within the petrous part of the temporal bone [8]. The intratemporal portion of the facial nerve branches into the greater petrosal nerve, the nerve to the stapedius muscle, and the chorda tympani nerve [9]. The extratemporal part of the facial nerve traverses through the stylomastoid foramen through the end of the posterior edge of the parotid gland, dividing into its terminal branches, i.e., temporal, zygomatic, buccal, mandibular, and cervical [10]. The terminal branches innervate a large portion of the face in motor and sensory components, such that it is a common site for iatrogenic complications involved with many common intraoperative procedures, particularly ones that involve mobilization or resection of associated anatomical structures [7,11,12].

Injury to the facial nerve or its branches has the potential to cause decreased quality of life and satisfaction as the degree of iatrogenic injury may result in partial or complete facial nerve paralysis [13,14]. The facial nerve is highly prone to injury due to its anatomical location, such that various strategies have been developed to decrease the incidence of nerve damage [15-17]. Of the various techniques developed to avoid injury, electromyography (EMG) has been widely used intraoperatively to monitor facial nerve function, determine the degree of injury, and predict postoperative weakness [18,19]. Here, we aim to analyze and review existing scientific literature in determining the role of intraoperative facial nerve monitoring with EMG and assess its efficacy in identifying and preventing damage to the facial nerve during various surgical procedures involved in the head and neck region.

## Review

Methodology

**Figure 2:**
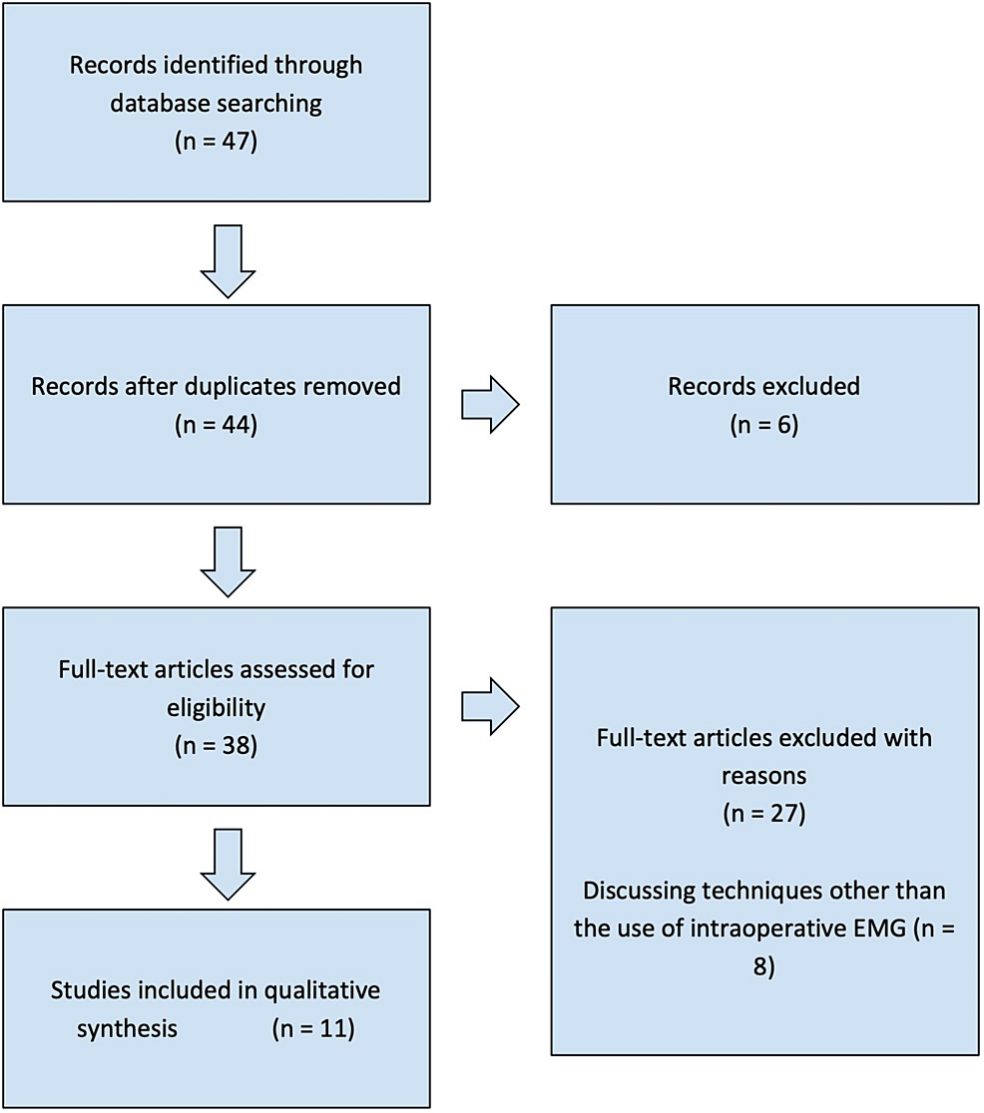
Preferred Reporting Items for Systematic Reviews and Meta-Analyses flowchart for articles included based on the inclusion and exclusion criteria. Of note, the eight articles excluded are within the 27 others excluded and separated out to highlight how other techniques were used in intraoperative nerve monitoring.

A systematic review was conducted from articles published between September 2006 to December 2022 from the MEDLINE/PubMed databases. Relevant terms such as “facial nerve injury,” “monitoring,” and “electromyography” were used to refine the initial search for iatrogenic facial nerve injury due to surgical intervention. Six records were excluded as full text could not be obtained, while an additional 27 met other exclusion criteria which were outside of date range, not published in English or having an English translation, or only peripherally related. Articles were subsequently coded based on the type of surgery performed with concurrent use of EMG as well as the results from intraoperative monitoring. Articles found were thoroughly analyzed based on relevance to the aims of this report. A total of 47 articles were found in relation to the use of intraoperative facial nerve monitoring, including studies to reduce the incidence and determine preventative measures to decrease nerve injury. After carefully considering the degree of information provided, a total of 11 articles were used to evaluate the use of EMG during various head and neck surgeries in decreasing the incidence of intraoperative facial nerve injury (Figure 2). This review was completed in accordance with Preferred Reporting Items for Systematic Reviews and Meta-Analyses guidelines.

Results

A total of 11 articles were selected to analyze the efficacy of intraoperative facial nerve monitoring in various head and neck surgeries [20-30]. From these 11 articles, Table 1 was created to examine the type of study done, the number of patients involved in each study, the specific head and neck pathology involved, and the respective intraoperative intervention performed.

**Table 1:** Summary of articles meeting the inclusion criteria regarding intraoperative facial nerve monitoring with electromyography.

Author and year	Type of study	Number of cases/patients	Surgical intervention
Chiesa-Estomba et al., 2021 [20]	Retrospective and prospective analysis	1,069 patients in the literature from articles published during 1970–2019	Primary parotidectomy
Meier et al., 2006 [21]	Retrospective analysis	45 patients	Primary parotidectomy
Haring et al., 2019 [22]	Retrospective case series	222 patients during 2004–2014	Primary parotidectomy
Liu et al., 2014 [23]	Cohort study with planned data collection	58 patients in 2004 and 2012 (28 monitored, 30 unmonitored)	Primary parotidectomy
Elsayed et al., 2021 [24]	Prospective study	43 patients in 2018	Vestibular schwannoma resection
Acioly et al., 2011 [25]	Prospective study	35 patients	Vestibular schwannoma resection
Ji et al., 2020 [26]	Randomized control trial	80 patients	Vestibular schwannoma resection
Hsieh et al., 2015 [27]	Retrospective study	654 patients during 1999–2014	Cochlear implant
Mandour et al., 2019 [28]	Retrospective study	307 patients during 2012–2017	Cochlear implant
Chiara et al., 2009 [29]	Retrospective study	7 patients	Lymphatic malformation surgery
Zhang et al., 2013 [30]	Retrospective study	8 patients	Vestibular schwannoma resection

Discussion

The 11 articles that noted the use of EMG were primarily divided based on the type of surgery performed. Overall, 36% of sources (n = 4) tested the efficiency of EMG in parotidectomy, 36% (n = 4) during vestibular schwannoma resection, 18% (n = 2) in cochlear implant surgery, and 9% (n = 1) during lymphatic malformation surgery. Many studies were retrospective (64%, n = 7), along with two prospective studies, one retrospective and prospective study analysis, one randomized control trial, and one cohort study. In the four studies done during parotidectomy, intraoperative facial nerve monitoring decreased the risk of weakness immediately postoperatively and long-term permanently [20-23]. Intraoperative facial nerve monitoring also decreased the duration of surgery, the severity of facial nerve palsy, and the average time of facial nerve paralysis recovery [23]. Similarly, for vestibular schwannoma resection cases, intraoperative facial nerve monitoring significantly preserved potential facial nerve function, as EMG proved to be effective in locating and protecting the facial nerve as well as preserving facial nerve function and decreasing paralysis risk [21,24,26,30]. Of note, EMG has been identified to have predictive value in postoperative nerve function and reduce the incidence of nerve injury in parotidectomy and vestibular schwannoma resection [22,25]. While limited studies exist on the use of intraoperative EMG in the surgical correction of cervicofacial lymphatic malformations, it has been shown that nerve mapping is useful in identifying and avoiding injury to the facial nerve [29,31]. For intraoperative facial nerve monitoring in cochlear implant studies, risks and benefits appeared equivocal as there was no significant effect on postoperative delayed facial palsy nor were there increased postoperative injuries in patients who did not have intraoperative facial nerve monitoring [27,28].

Intraoperative facial nerve monitoring is a valuable resource in locating and mapping out the facial nerve to avoid intraoperative injury and postoperative paralysis. In identifying the course of the nerve, intraoperative facial nerve monitoring allows for the identification of the nerve course, adequate protection, and the preservation of facial nerve function [32-34]. Facial nerve protection results in decreased manipulation of the nerve, reducing numbness associated with nerve injury, thus increasing patient satisfaction [35,36]. Despite its benefits, intraoperative facial nerve monitoring is limited by its ability to accurately prognosticate intraoperative nerve injury [21,37,38]. Due to the decreased efficacy and nonsignificant findings with intraoperative facial nerve monitoring during cochlear implants, the value of intraoperative facial nerve monitoring can also depend on the location of usage and the nature of surgical intervention [39-41]. For example, facial palsy following cochlear implants is rare, with incidence ranging from 0.62% to 0.73% in patients with intraoperative facial nerve monitoring versus 0.72% in patients without intraoperative facial nerve monitoring [27,28]. In contrast to cochlear implants, facial nerve injury during general otologic procedures is around 17% [42]. Future studies are needed to determine if intraoperative facial nerve monitoring can be useful in maintaining the integrity of the facial nerve in other otologic procedures and thus improving surgical outcomes.

EMG and its research have been relatively emergent; however, newer technology and techniques have also been shown to reduce intraoperative facial nerve injury and better predict nerve injury during surgical interventions. For example, in a recent retrospective study of parotidectomies, magnetic resonance imaging with three-dimensional double-echo steady-state (DESS protocol) and water excitation sequence was a superior method in localizing the facial nerve intraoperatively [43-45]. With a mixture of deep and superficial lesions, direct visualization of the intraparotid facial nerve was consistently achieved leading to increased diagnostic accuracy, sensitivity, specificity, and positive predictive value [46,47]. Limitations to widely using this protocol are the availability of magnetic resonance imaging equipment, technical expertise in the interpretation of the images, and proprietary licensing of the protocol among others [48-50]. Further research is necessary to validate the findings from this study and other new techniques aimed to support and/or replace EMG techniques.

## Conclusions

Overall, intraoperative facial nerve monitoring using EMG as an adjunct during surgery may reduce the risk of iatrogenic injury by locating and protecting the facial nerve, but additional studies must be performed to determine the degree of long-term patient satisfaction and quality of life achieved in the setting of intraoperative facial nerve monitoring. Based on the current evidence, surgical settings may determine the efficacy and use of intraoperative facial nerve monitoring in decreasing the incidence of facial nerve weakness and paralysis. Many new techniques should also be considered in understanding the role of other techniques in decreasing the incidence of intraoperative facial nerve injury. Increasing incidences of facial nerve injury seen with various head and neck surgeries support the importance of determining the role of EMG in intraoperative facial nerve monitoring and the need for a predictive intraoperative tool.
